# LPO-YOLOv5s: A Lightweight Pouring Robot Object Detection Algorithm

**DOI:** 10.3390/s23146399

**Published:** 2023-07-14

**Authors:** Kanghui Zhao, Biaoxiong Xie, Xingang Miao, Jianqiang Xia

**Affiliations:** 1 Beijing Engineering Research Center of Monitoring for Construction Safety, Beijing University of Civil Engineering and Architecture, Beijing 100044, China; 2108550022112@stu.bucea.edu.cn (K.Z.);; 2 Anhui Hengli Additive Manufacturing Technology Co., Ltd., Wuhu 241200, China

**Keywords:** casting process, object detection, YOLOv5s, DSIFM

## Abstract

The casting process involves pouring molten metal into a mold cavity. Currently, traditional object detection algorithms exhibit a low accuracy and are rarely used. An object detection model based on deep learning requires a large amount of memory and poses challenges in the deployment and resource allocation for resource limited pouring robots. To address the accurate identification and localization of pouring holes with limited resources, this paper designs a lightweight pouring robot hole detection algorithm named LPO-YOLOv5s, based on YOLOv5s. First, the MobileNetv3 network is introduced as a feature extraction network, to reduce model complexity and the number of parameters. Second, a depthwise separable information fusion module (DSIFM) is designed, and a lightweight operator called CARAFE is employed for feature upsampling, to enhance the feature extraction capability of the network. Finally, a dynamic head (DyHead) is adopted during the network prediction stage, to improve the detection performance. Extensive experiments were conducted on a pouring hole dataset, to evaluate the proposed method. Compared to YOLOv5s, our LPO-YOLOv5s algorithm reduces the parameter size by 45% and decreases computational costs by 55%, while sacrificing only 0.1% of mean average precision (mAP). The model size is only 7.74 MB, fulfilling the deployment requirements for pouring robots.

## 1. Introduction

Casting is the basic process of the equipment manufacturing industry, and pouring is a crucial step in the casting process.Detection of pouring hole positions belongs to the object detection task in computer vision, which finds application in various industrial fields. Traditional object detection algorithms typically rely on manually designed feature extractors, such as SIFT [1], HOG [2], and others. However, these handcrafted features may not be suitable for different tasks and datasets, necessitating frequent adjustments and modifications. This increases the complexity and labor costs of the algorithms. Furthermore, traditional object detection algorithms are relatively slow and fail to meet real-time requirements. They also exhibit insensitivity to complex scenes and target occlusions, often resulting in inaccurate detection of partial targets or difficulties in distinguishing between targets. In contrast, deep-learning-based object detection algorithms have gained wide adoption in various intelligent robotics applications, due to their higher accuracy and faster speed. In deep learning, object detection can be categorized into two types: two-stage detection algorithms, and one-stage detection algorithms [3]. Two-stage detection algorithms generally achieve a higher localization and target recognition accuracy, but they are slower in terms of detection speed. Common two-stage algorithms include R-CNN [4], Fast R-CNN [5], Faster R-CNN [6], and Mask R-CNN [7]. In the field of unmanned aerial vehicles (UAVs), Yun et al. [8] proposed an improved algorithm for landslide detection in UAV imagery based on Mask R-CNN. By introducing attention mechanisms and modifying the feature pyramid network’s structure, they were able to enhance the accuracy of landslide information extraction. On the other hand, one-stage algorithms offer a faster detection speed but may have a lower detection accuracy, such as SSD [9] and YOLO [10,11,12]. In the transportation domain, Lai et al. [13] developed a small traffic sign detection network (STC-YOLO) based on YOLOv5, for complex environments. By introducing a multi-head attention module and improving the loss function, they achieved a 5.9% improvement in mean average precision (mAP), yielding promising results. For the detection of small objects, Xiao et al. [14] proposed a context-enhanced and feature-refined network specifically designed for detecting small objects. Additionally, in many practical studies, the large model size of deep learning poses efficiency challenges when running on resource-limited embedded devices. Consequently, researchers have focused on lightweight model designs. Prominent lightweight models include SqueezeNet [15], MobileNet [16,17,18], ShuffleNet [19,20], and GhostNet [21], which have a reduced number of parameters and computational complexity to some extent without significant performance degradation. Jia et al. [22] proposed a lightweight and high-performance rice pest identification model, by replacing the backbone network of YOLOv7 with MobileNetv3 and combining this with an attention mechanism. It is worth mentioning that compared to lightweight networks such as ShuffleNetv2, MobileNetv3 can better balance efficiency and accuracy when completing real-world image classification tasks on mobile terminals and has therefore been widely adopted [23].

Sampling on feature maps is a very important step in object detection. The CARAFE [24] operator has a large receptive field and sufficient semantic information, without adding too many parameters, and its performance is better than transpose convolution [25] and other upsampling operations. Zhang et al. proposed a novel quad feature pyramid network (Quad-FPN) combined with a deformable convolutional FPN (DE CO FPN), content aware feature reassembly FPN (CA FR FPN), a path aggregation space attention FPN (PA SA FPN), and a balance scale global attention FPN (BS GA FPN), to achieve real-time monitoring of ship images [26].

At present, there are two main types of head modules for object detection: coupling heads, and decoupling heads. Among them, the detection effect of the decoupling head is higher than that of the coupling head in most cases. Muralidhara et al. [27] increased the upper limit of current video object detection methods by introducing DyHead detection heads into faster R-CNN, combining scale, space, and task aware attention, and achieving good results.

In recent years, with the rapid development of deep learning, more and more researchers are committed to research in the field of object detection (i.e., backbone, neck, and head). Li et al. proposed omnidimensional dynamic convolution (ODConv) [28], ODConv is a more generalized, yet elegant, dynamic convolution design. It utilizes novel multidimensional attention mechanisms and parallel strategies, to learn the complementary attention of convolutional kernels in all dimensions of the kernel space. Yu et al. proposed PoolFormer [29] by replacing the attention module in transformers with a spatial pooling operation, which achieved a good detection performance. Yang et al. proposed focal modulation networks (FocalNet) [30]; this network can modulate automatically and gradually converge to the object area that induces the recognition of the category. Yu et al. proposed a new network called InceptionNeXt [31] by combining inception with ConvNeXt, which not only had a high throughput but also maintained competitive performance. Generalized FPN [32] refines and integrates high-level semantic features and low-level spatial features. Based on GFPN, Xu et al. [33] proposed a novel Efficient-RepGFPN to achieve the design of real-time object detection. Li et al. designed a new neck network, named slim neck [34], to achieve a higher computational cost-effectiveness of detectors. Zhuang et al. proposed a novel task-specific context decoupling (TSCODE) [35] head, which further disentangles the feature encoding for classification and positioning tasks. Li et al. [36] adopted a hybrid-channel strategy, to build a more efficient decoupled head, which accelerated the inference speed of the network. However, a key issue that almost all of the above modules face is the significant increase in the number of network parameters and computational complexity, making them unsuitable for lightweight research on networks.

Regrettably, there has been no research on the visual recognition of pouring robots conducted by researchers. Therefore, the purpose of this study was to design a lightweight object detection algorithm suitable for pouring robots, which could be used to solve the problem of large-scale neural network models deployed in devices with limited resources and seizing other logical and peripheral resources. Inspired by the aforementioned works, this paper introduces the LPO-YOLOv5s algorithm, which stands for lightweight pouring hole detection algorithm based on YOLOv5s. To achieve this, we propose utilizing the MobileNetv3 network as the backbone network. By doing so, we can significantly reduce the model’s parameters and computational complexity. However, reducing parameters often leads to a certain loss in performance. In order to minimize this loss, we designed a depthwise separable information fusion module (DSIFM) and incorporated it between the backbone layer and the neck layer of the algorithm. This addition does not result in a significant increase in parameters. In the neck network, we also considered using CARAFE, due to its lightweight features. Additionally, DyHead was employed in the network prediction stage to fine-tune the model’s performance. The lightweight model proposed in this paper achieved excellent detection results, with fewer parameters and a smaller overall size.

The following are the main contributions of this article:

1. A lightweight object detection algorithm for pouring tasks is proposed, with MobileNetv3 as the backbone network, greatly reducing the parameter number and computational complexity;
2. A DSIFM module is designed, to enable the network to obtain different levels of receptive field, and enrich and fuse the contextual feature information, so as to carry out effective feature extraction;
3. During the upsampling process, a CARAFE operator is used to better focus on the relevant point information in the local area and obtain stronger semantic information;
4. In the model detection phase, a plug and play DyHead detection head is used to improve the detection performance of the algorithm;
5. A dataset of pouring hole locations has been established, to provide assistance and a reference for future research.

## 2. Methods

### 2.1. Basic Module

**Figure 1:**
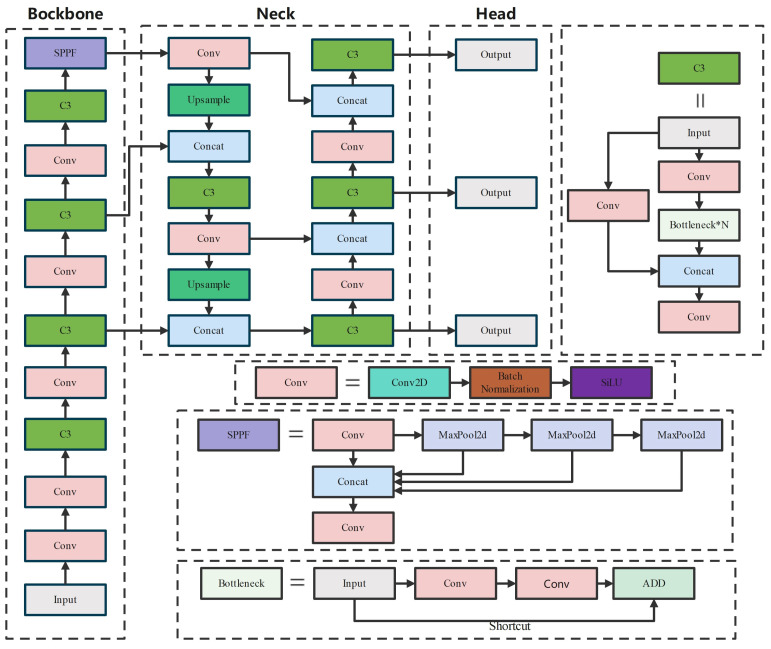
Structures of YOLOv5s network.

YOLOv5 is a one-stage object detection algorithm that utilizes multiple layers of convolutional neural networks to extract the position and category information of objects. The network structure of YOLOv5 consists of four parts: input, backbone, neck, and prediction. The input part preprocesses the dataset and includes operations such as mosaic data augmentation, adaptive anchor box calculation, and adaptive image scaling. The backbone is comprised of Conv modules and C3 modules. The Conv module includes Conv2D, Batch Normalization, and SiLU activation function. The C3 module consists of three standard convolutions and multiple bottleneck modules, which are interconnected through residual connections to extract features from the feature map. The SPPF module, located at the end of the backbone, is based on the SPP [37] (spatial pyramid pooling) module and aims to extract high-level semantic features by performing multiple max pooling operations. The neck part adopts the structures of a feature pyramid network (FPN) [38] and pyramid attention network (PAN) [39]. The FPN propagates high-level semantic features in a top-down manner, through upsampling and fusion, while the PAN conveys strong localization features of shallow-level feature maps in a bottom-up manner. By combining FPN and PAN, the network obtains both localization and semantic information, enhancing the feature fusion capability of the network. In the prediction phase, all generated prediction boxes are filtered using the complete intersection over union (CIoU-loss) [40] and non-maximum suppression (NMS) [41] algorithms. The prediction boxes with the highest confidence scores are outputted, completing the object detection process. The specific flowchart is shown in Figure 1.

### 2.2. LPO-YOLOv5s

**Figure 2:**
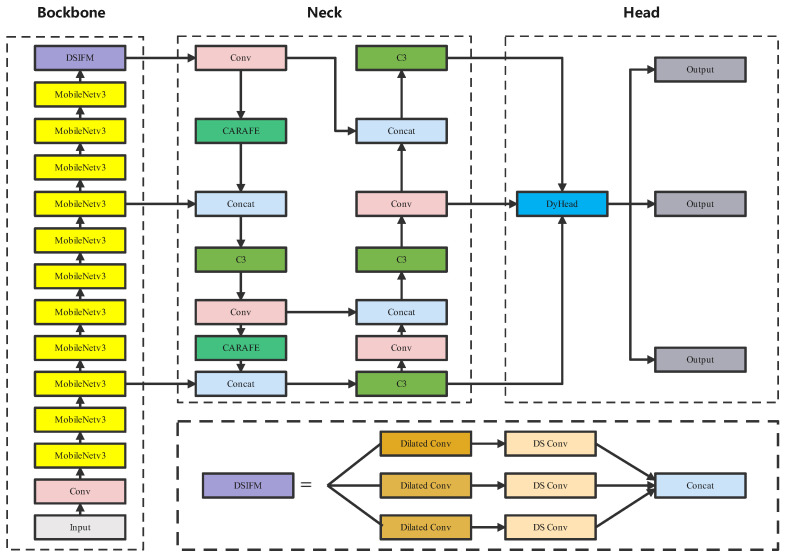
Structure of the LPO-YOLOv5s network.

The LPO-YOLOv5 algorithm represents an improvement over YOLOv5. The overall structure of the network is similar, comprising an input, backbone network, neck network, and head network, as depicted in Figure 2. To achieve a lightweight model and reduce the parameter quantity and network complexity, we employ a lightweight MobileNetv3 network in the backbone network for feature extraction. Addressing the issue of imbalanced information distribution between high-level and low-level feature maps, we introduce DSIFM to replace the SPPF module. In the neck network, we utilize the CARAFE module to effectively aggregate contextual information. Furthermore, the DyHead detection head in the head network serves as a plug-and-play module for detecting different perspectives, thereby improving the detection performance of pouring hole positions.

#### 2.2.1. Lightweight Backbone: MobileNetv3

The MobileNetv3 network retains the effective depthwise separable convolution of MobileNetv1 and the linear bottleneck inverted residual structure of MobileNetv2. Additionally, it introduces a lightweight attention module called an SE structure. Moreover, the network incorporates a novel non-linear activation function called h-swish, which combines with the ReLU activation function to reduce memory access, improve network accuracy, and minimize latency costs.

**Figure 3:**
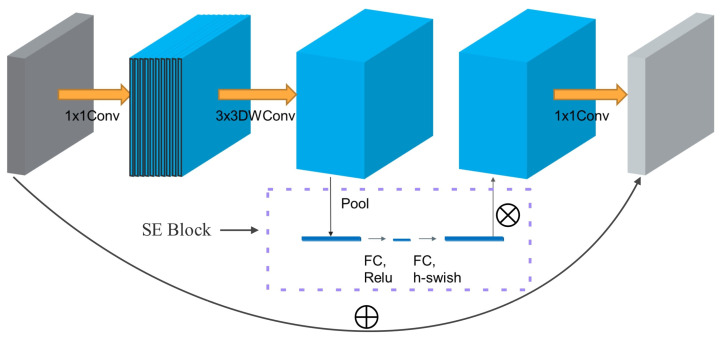
Structure of the lightweight backbone: MobileNetv3.

In Figure 3, the module begins by expanding the input features through a 1 × 1 convolution, to enhance the information content of the image. Next, the expanded features undergo a 3 × 3 convolution for depthwise separable convolution, which reduces the parameter computation. The resulting features from the depthwise separable convolution are then passed through the SE attention module. Within this module, the features are globally average pooled and processed by two fully connected layers, automatically assigning weights to the feature maps. This weight allocation amplifies valuable feature channels, while suppressing irrelevant ones. It is worth noting that the activation function for the first fully connected layer is ReLU, while the second fully connected layer employs h-swish. Finally, the reweighted features are dimensionally reduced through a 1 × 1 convolution and added back to the original input feature map, resulting in a new feature map.

#### 2.2.2. CARAFE: Lightweight Upsampling Operator

Feature upsampling plays a critical role in the feature pyramid, particularly in object detection tasks. YOLOv5 incorporates the nearest-neighbor interpolation method [42] as its feature upsampling operator. This method employs grayscale values of the nearest pixels to guide the upsampling process, utilizing the same upsampling kernel for every position in the feature map. While this algorithm is computationally efficient and fast, it neglects the influence of neighboring pixels, resulting in noticeable discontinuities in the resampled grayscale values and a significant loss of image features. To address these limitations, CARAFE introduces a universal lightweight upsampling operator that performs upsampling based on the input content, incorporating a larger receptive field, to efficiently aggregate contextual information. CARAFE is lightweight, requires no additional parameters or computational cost, and provides increased processing speeds.

**Figure 4:**
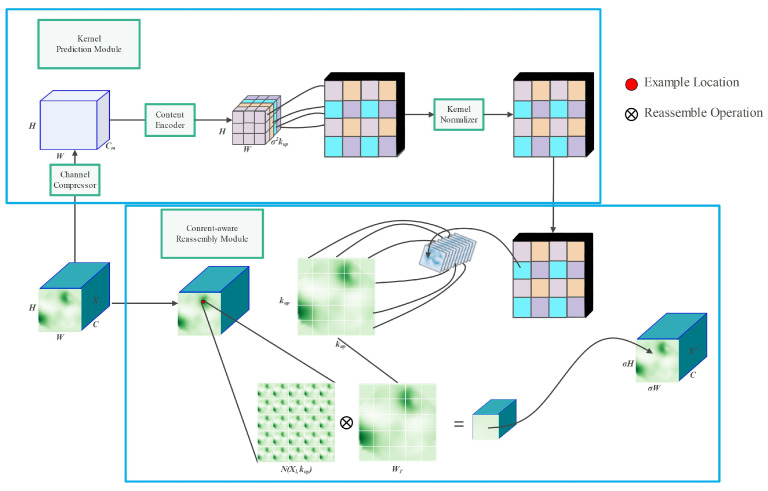
Lightweight upsampling operator: CARAFE. Note: H, height of the pouring hole feature map; W, width of the pouring hole feature map; $C_{m}$, length of the compressed pouring hole feature map; C, length of the original pouring hole feature map; X, original pouring hole feature map; σ, up-sampling multiplier; $k_{up}$, reorganization kernel size; $X^{\prime }$, reorganization pouring hole feature map; $W_{l^{\prime }}$, feature reorganization kernel; N, square region; $X_{l}$, feature map at l.

Figure 4 illustrates the module structure of CARAFE. Assuming the size of the input feature map is C × H × W, the upsampling factor is σ, and the size of the upsampling kernel is $k_{up}$. First, the given feature map is compressed along the channel dimension using a 1 × 1 convolution, resulting in a compressed feature map of size H × W × $C_{m}$. Further channel compression reduces the computational burden of the network and accelerates the processing speed. Next, a convolutional layer with a size of $k_{encoder}$ × $k_{encoder}$ is used to encode and predict the upsampling kernel based on the compressed feature map. The input channel is $C_{m}$, and the output channel is $\sigma^{2}k_{up}^{2}$. The predicted upsampling kernel is then unfolded in the spatial dimension, resulting in an upsampling convolutional kernel of shape σH × σW × $k_{up}^{2}$. Finally, the Softmax function is applied to normalize the upsampling kernel, ensuring that the sum of its weights is 1.

Through the action of each recombined convolutional kernel $W_{l^{\prime }}$, the content-aware reassembly module recombines features within local regions using a weighted summation operator. The recombination process for the target position *l*′ and the corresponding square region N($X_{l^{\prime }}$,$k_{up}$) centered at *l* = (*i*,*j*) is shown in Equation (1), where *r* = ⌊$k_{up}$/2⌋.

(1) $$X_{l^{\prime }}^{\prime }=\sum_{n=-r}^{r}\sum_{m=-r}^{r}W_{l^{\prime }\left(n,m\right)}\cdot X_{\left(i+n,j+m\right)}$$

The feature map after the CARAFE operator can better focus on relevant point information within local regions compared to the nearest-neighbor interpolation sampling method, enabling the capture of stronger semantic information.

#### 2.2.3. Depthwise Separable Information Fusion Module: DSIFM

**Figure 5:**
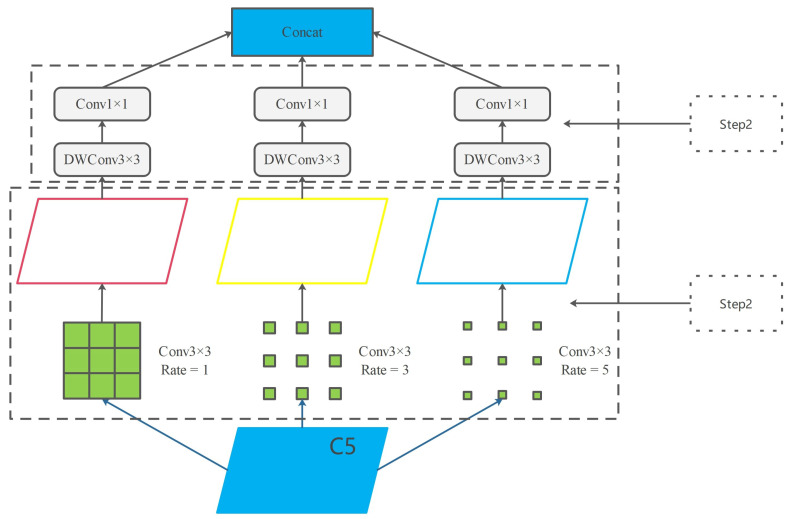
The overall framework of the DSIFM. This module mainly consists of two steps: a multi-scale dilated convolution module, and depth separable convolution module.

Background contextual information plays a crucial role in object detection tasks and should also be considered in the pouring task. In the benchmark model YOLOv5, the SPPF module connects the backbone feature extraction network and the neck feature fusion network, partially addressing the challenge of the network’s difficulty in extracting high-level semantic features. However, the max pooling layer in the module leads to the loss of detailed information in the feature map. This lost information cannot be recovered through subsequent upsampling operations, making it challenging for the network to acquire sufficient contextual information. As a result, the algorithm’s performance in complex tasks is suboptimal. To overcome this limitation, we propose a deep separable information fusion module (DSIFM) to replace the SPPF module. Our DSIFM aims to obtain contextual information with different receptive fields, and a model structure diagram is shown in Figure 5.

To enrich the contextual feature information and facilitate effective feature extraction, we applied dilated convolution operations to the feature maps that underwent five downsampling operations. We used dilation rates of 1, 3, and 5, as features with both large and small receptive fields, to complement each other. By incorporating different dilation rates, the object detection network can acquire multiple levels of receptive fields. This approach enhances contextual feature information. Moreover, dilated convolution introduces zeros between convolutional kernels, resulting in a relatively smaller number of additional parameters. This aligns with our objective of designing a lightweight network. Then, the feature maps outputted from the dilated convolution module were each subjected to a depthwise separable convolution and finally combined for enhanced network robustness.

#### 2.2.4. Dynamic Head: Unifying with Attentions

Due to the variations in pouring tasks, the size of pouring holes can differ significantly. Consequently, the detection head of the network must possess the capability to detect pouring holes of varying scales. Furthermore, considering the robot’s positional movement during operation, the detection head needs to incorporate spatial perception. To address these challenges, we introduced DyHead as the detection head algorithm during the network’s prediction stage. DyHead is a multi-attention unified detection head method proposed by Dai et al. [43]. It treats the input of the backbone network as distinct attention mechanisms deployed at the scale level, spatial level, and channel level. This approach achieves a unified attention mechanism for the detection head. Whether used in a one-stage or two-stage object detection algorithm, DyHead, as a plug-and-play lightweight module, can be embedded to enhance the detection performance of the algorithm.

Before applying the DyHead detection head, it is necessary to scale the feature pyramid outputted by the backbone network to a consistent scale. The feature maps of the scaled pyramid can be represented as a four-dimensional tensor $F\in R^{L\times H\times W\times C}$, where *L* denotes the number of levels in the feature pyramid, and *H* and *W* represent the height and width of the feature map, respectively. The channel dimension is denoted by *C*, indicating the number of channels in the feature map. As H and W represent spatial features, they are replaced with S, defined as S = H × W. This abstraction allows us to view the feature map as a three-dimensional tensor, with dimensions L, S, and C focusing on scale perception, spatial perception, and channel perception, respectively.

**Figure 6:**
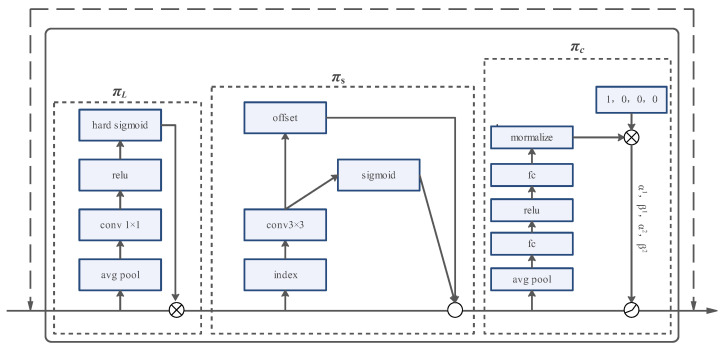
A specific introduction to DyHead.

Figure 6 illustrates the principle of the DyHead. The feature maps undergo mapping and sequentially pass through the scale perception module ($\pi_{L}$), spatial perception module ($\pi_{S}$), and task perception module ($\pi_{C}$), to achieve a unified attention mechanism for the detection head. In the scale perception module, the L dimension of the feature map undergoes global average pooling, followed by feature extraction using a 1 × 1 convolutional layer and ReLU activation function. The result is then passed through a hard sigmoid activation function and element-wise multiplied with the input feature map, concluding the scale perception module. For the spatial perception module, the input tensor is processed using a 3 × 3 convolutional layer, to obtain the offset values of the feature map, as well as the weight terms of the feature map offsets. Spatial perception is achieved through the utilization of deformable convolutional [44]. In the task perception module, the tensor undergoes global average pooling on the L × S dimension to reduce the dimensionality and parameter count. It then proceeds through two fully connected layers and a normalization layer. Dynamic ReLU [45] is applied, which outputs different channel values based on different tasks, completing the task perception of the feature map.

## 3. Expression Results

### 3.1. Experimental Setting

This study utilized Python version 3.8, PyTorch version 1.11.0, CUDA version 11.2, and a GeForce RTX 3060 VENTUS 2X 12G OC graphics card. The chosen hyperparameters included hyp.scratch-low, a batch size of 32, and 300 rounds of training. In order to ensure the rigor and smooth progress of the experiment, the pre-training weights and other parameters all adopted the default values of YOLOv5s.

### 3.2. Experimental Dataset

#### 3.2.1. Data Acquisition

**Figure 7:**
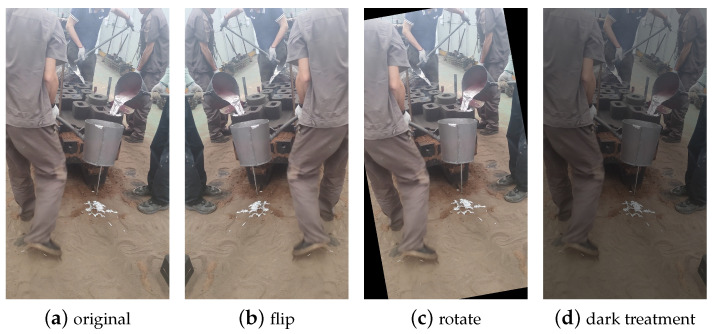
Data set of hole location detection in the pouring environment.

Due to the limited number of researchers studying pour hole detection, a publicly available complete dataset is currently unavailable. Therefore, all the data in this study were captured in the natural working environment of the pouring workshop at Anhui Hengli Additive Manufacturing Technology Co., Ltd., in Wuhu City, Anhui Province, China, using a smartphone. To ensure the quality and effectiveness of the dataset, we aimed to cover different shooting scenarios as comprehensively as possible. During the data collection process, we observed the impact of lighting conditions on image quality. Hence, we made efforts to include various lighting conditions in the collection, encompassing both natural and artificial light. Additionally, we considered the diversity of shooting angles and distances, to ensure sufficient coverage in the dataset. As pouring tasks in the factory may involve unrelated personnel obstructing the pour holes, we also incorporated data with partially obscured pour holes, to enhance the dataset’s robustness. Through a series of data augmentation operations, such as translation and flipping, the final dataset consisted of 2790 images. These images were divided into training, testing, and validation sets in an 8:1:1 ratio. The resolution of the data ranged from 1080 pixels × 1920 pixels to 2080 pixels × 4608 pixels. Sample images from the dataset are shown in Figure 7.

#### 3.2.2. Data Annotation

In addition to dataset acquisition, the annotation task is also crucial. We employed the Labelimg tool for the manual annotation of the dataset. Labelimg can generate annotation data in two formats: VOC format and YOLO format. For the convenience of experimentation, all image data annotations were conducted with the YOLO format. Through manual annotation, we ensured that each photo accurately marked the position and size of the pouring hole. Considering and addressing all these details contributed to the quality and reliability of the dataset, enhancing the accuracy and generalization capability of the model, and providing effective data support for the pouring hole detection task.

### 3.3. Evaluation Metrics

This paper evaluated the LPO-YOLOv5s model using widely recognized evaluation metrics in the field of object detection. The evaluation metrics employed included the precision rate (*P*), recall rate (*R*), mAP, F1-score, and weight size. Since this article presents a lightweight object detection algorithm, the parameter count was the most crucial metric. The definitions of the various evaluation metrics are as follows:

(2) $$P=\frac{TP}{TP+FP}$$

(3) $$R=\frac{TP}{TP+FN}$$

In Equations (2) and (3), TP represents the number of correctly detected hole positions, where the detection results and the labels in the images matched. Similarly, FP indicates the number of false detections, where the detection results identified hole positions, but the ground truth labels were backgrounds. FN represents the number of missed hole positions, where the model’s detection results identified backgrounds, but the model labeled them as hole positions, indicating the rate of missed hole positions.

(4) $$F1\text{-}\mathrm{score}=2\times \frac{P\times R}{P+R}$$

In Equation (4), the *F*1-*score* is used to balance the weights of *P* and *R*, representing the value when both reach their highest points simultaneously.

(5) $$AP=\int_{0}^{1}P\left(R\right)dR$$

(6) $$mAP=\frac{1}{N}\sum_{i=1}^{N}AP_{i}$$

Here, *AP* represents the area under the *P-R* curve, *N* represents the total number of classes, and *mAP* is the average value of *AP* across all classes. In this paper, *mAP* is reported as mAP@0.5, indicating the average precision when the IoU threshold was set to 0.5, In the experiments, a higher *mAP* value signified a better model performance, indicating the algorithm’s effectiveness.

### 3.4. Results

**Figure 8:**
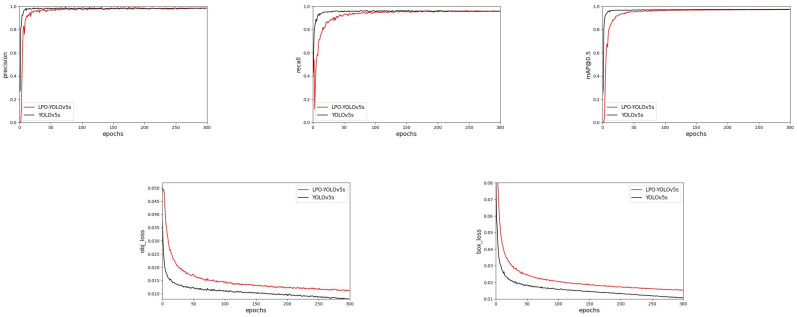
The model training results for LPO-YOLOv5s and YOLOv5s.

This paper conducted numerous experiments using the pouring hole detection dataset, and the metrics obtained during the model training process are shown in Figure 8.

From Figure 8, it is evident that, as the number of model training iterations was increased, both the accuracy, recall rate, and mAP@0.5 value of the original YOLOv5s algorithm and the LPO-YOLOv5s algorithm gradually improved. Although replacing the backbone network resulted in significant fluctuations and a slower convergence speed of the LPO-YOLOv5s algorithm in the first 50 epochs, when the number of training iterations reached 100 epochs, the metrics of both algorithms gradually stabilized and almost converged to the same value. We also found that, compared to the yolov5s algorithm, our method had a higher median value in the model loss curve, but ultimately converged to a relatively small value, which was in line with the experimental expectations.

**Figure 9:**
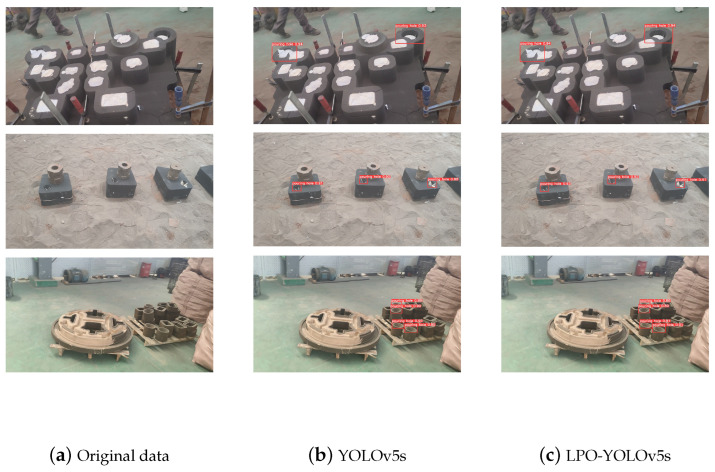
The detection performance of the two algorithms before and after improvement.

Additionally, according to Table 1, we can see that our algorithm, compared to YOLOv5s, experienced only a slight decrease of 0.1% in mAP@0.5, while reducing the parameter count by 45% and lowering the computational complexity by 55%. This makes it easier to deploy in hardware and meets the requirements for pouring robots. The detection results of the two algorithms are shown in the Figure 9.

#### 3.4.1. Comparative Experiment

To further demonstrate the performance advantages of the LPO-YOLOv5s algorithm, this paper conducted experimental comparisons on the pouring hole dataset using several mainstream object detection algorithms. Since the algorithm proposed in this paper is an improvement on one-stage algorithms, only faster R-CNN, a two-stage algorithm, was selected for comparison, while the rest were single-stage detection models such as SSD, YOLOX [46], YOLOv7 [47], YOLOv8, and so on. The experimental results are shown in Table 2.

According to Table 2, it can be observed that the proposed algorithm achieved the highest precision among the algorithms in the table, with a value of 98.2%. This precision value was 10.1% higher than that of the Faster R-CNN algorithm and 0.4% higher than that of the YOLOv5s algorithm. Moreover, the proposed algorithm also performed well in terms of the F1 score, reaching 0.97, which was the highest value among all the algorithms. The faster R-CNN algorithm had the highest recall rate among all the algorithms. However, due to its large parameter size and lower values in other metrics, it did not meet the requirements for pouring robot operation. It is worth noting that the YOLOX-s algorithm had the highest mAP@0.5 value. However, its model size was relatively large, making it unsuitable for deployment in resource-limited embedded devices. Similarly, YOLOv8 had the fastest inference speed, reaching 131 FPS, but its model size was about three times that of the algorithm in this paper. Additionally, during the experiments, we found that the recall rate of the proposed method was relatively low compared to the other state-of-the-art algorithms, and there was no significant improvement compared to the original YOLOv5s. However, in the context of pouring tasks, precision is of greater importance. Due to the nature of pouring tasks involving human operators, a slight decrease in recall rate would not significantly affect the progress of pouring tasks. On the contrary, a higher precision value indicates a more accurate detection and better performance in completing pouring tasks.

#### 3.4.2. Ablation Experiment

To verify the effectiveness of each module of the proposed LPO-YOLOv5s algorithm in pouring hole detection, this study conducted ablation experiments under the same experimental conditions, using the precision and mAP@0.5 as evaluation metrics. From Table 3, it can be observed that the MobileNetv3 module was primarily introduced to lighten the model, which indeed resulted in some performance loss. However, with the integration of other modules, the performance of the improved model gradually improved. Among them, the DSIFM module improved the accuracy of the model by 0.4%, while the CARAFE module primarily affected the recall rate of the model. Finally, it was found that the addition of the DyHead module on top of these improvements made the model even more prominent, with significant improvements in the precision and mAP@0.5 values. The experimental results fully demonstrate the effectiveness of the designed LPO-YOLOv5s algorithm presented in this paper.

## 4. Conclusions

The total number of foundries in China has exceeded 20,000, with an average annual production of 1777 tons of castings per factory, which is significantly lower than the top-ranked country Germany, producing 8818 tons. China ranks last among the top-ten countries in global casting production. This is primarily due to the low level of automation in the foundry industry. To tackle this challenge, as well as enhance the intelligence and meet the demands of digitized factories, this paper proposed a lightweight pouring hole detection algorithm for pouring robots. In terms of reducing parameters, we introduced the MobileNetV3 network to replace the backbone of YOLOv5s. However, through experiments, we found that the performance of the algorithm declined to a certain extent. Therefore, between the backbone network and the neck network of YOLOv5s, we designed a DSIFM module, to strengthen the information fusion between the network layers. Experiments proved that the DSIFM module improved the detection accuracy, and the CARAFE upsampling operator improved the algorithm’s recall rate. At the end of the algorithm, a DyHead attention detection head was used, and the detection advantages of the algorithm in this paper were shown through testing with a pouring dataset. While minimizing performance loss, the algorithm achieved significant reductions in parameters and computational complexity, with the model size being only 7.74 MB, a 43% reduction compared to the unimproved scale. Through experimental comparisons with other algorithms, the proposed algorithm achieved a highest accuracy of 98.2%, meeting the requirements for pouring robots, in terms of both detection accuracy and parameter scale. In the future, we will further optimize the pouring robot to accurately identify and locate targets. At the same time, we will also consider improving the robot’s autonomous navigation ability for independently planning paths, to accelerate the automation process of the casting industry.

## Figures and Tables

**Table 1:** The results of the LPO-YOLOv5s and YOLOv5s models in the validation.

Model	P	R	F1	mAP@0.5	GFLOPs	Parameters	FPS	Weight Size
YOLOv5s	97.8%	96.4%	0.97	97.3%	15.8	7,012,822	91	13.6 (MB)
Ours	98.2%	96.0%	0.97	97.2%	7.0	3,874,924	80	7.74 (MB)

**Table 2:** Comparative experimental results of the various models on the hole detection dataset.

Model	P	R	F1	mAP@0.5	FPS	Weight Size
SSD	96.8%	97.7%	0.87	95.7%	62	90.6 (MB)
Faster-RCNN	88.1%	98.3%	0.88	98.1%	11	108 (MB)
YOLOv3	96.9%	92.4%	0.95	97.3%	63	235 (MB)
YOLOX-S	96.8%	95.9%	0.96	98.7%	82	34.3 (MB)
YOLOv7-tiny	97.8%	95.3%	0.96	96.3%	85	11.6 (MB)
YOLOv8s	97.4%	97.0%	0.97	97.2%	131	21.4 (MB)
Ours	98.2%	96.0%	0.97	97.2%	80	7.74 (MB)

**Table 3:** The results of the ablation experiment.

Model	MobileNetv3	DSIFM	CARAFE	DyHead	Precision	mAP@0.5
YOLOv5s	×	×	×	×	0.978	0.973
A	*√*	×	×	×	0.974	0.965
B	*√*	*√*	×	×	0.979	0.966
C	*√*	*√*	*√*	×	0.979	0.968
D	*√*	*√*	*√*	*√*	0.982	0.972

## Data Availability

Not applicable.
